# 

**Published:** 1917-09-22

**Authors:** 


The Fishmongers' Company have made, amongst others, the following grants: Twenty-four guineas to the Children's Hospital for Hip Disease, Sevenoaks, £25 to Tooting Military Hospital (towards a fund for providing employment for sick and wounded soldiers confined to bed), £21 to the Soldiers' and Seamen's Home, Gibraltar, £105 to the French Red Cross, £250 to the British Red Cross, £21 to St. Mary's Hospital for Women and Children, and £52 10s. to the Royal National Hospital for Consumption, Ventnor.

### The Poet's Contribution.

Readers of the delightful stories and poetry of Private Patrick MacGill will be interested to hear that from the proceeds of the public readings of his works by his wife, Mrs. Patrick MacGill, two beds have been endowed in the King Edward Memorial Hospital, Ealing. They are named "The London Irish," after her husband's regiment, and "The Patrick MacGill" beds. Over the latter appear these lines, written by the soldier-poet:—

"This is your bed, and I hope you'll be gay in it;
Peace and contentment be yours while you stay in it;
This wish of mine will be yours, I've no doubt of it;
Soft be your bed, but I wish you well out of it."

### Matrons' and Sisters' Appointments.

Astoria Hospital, Paris.—Miss C. C. du Sautoy has been appointed matron. She was trained at Guy's Hospital, and worked for several years under the Queen Victoria Jubilee Nurses' Institute, being inspector for Wales and later county superintendent for Somerset. Since the war she has been matron of the Red Cross Hospital at Standish, Glos., was later on the staff of the Ulster Volunteer Force Hospital at Lyons, and for the past six months has been sister at the Ambulance de l'Océan, La Panne.

Huddersfield Royal Infirmary.—Miss Madge Abram has been appointed assistant matron and home sister. She was trained at the Manchester Royal Infirmary, where she was later surgical sister. She has since been night sister at Harborne Hall Auxiliary Hospital, Birmingham, and later sister-in-charge of the new branch of that hospital.

Grata Quies Auxiliary Hospital, Branksome Park, Bournemouth.—Miss Margaret Webster has been appointed night sister. She was trained at the Metropolitan Hospital, London, where she was later night sister.

Manchester Children's Hospital, Pendlebury.—Miss Doris King has been appointed sister at this hospital, where she took part of her training. She also trained at Guy's Hospital, and was sister at Alexandra Hospital, Queen Square.

Stoke-on-Trent Union Hospital, near Newcastle-under-Lyme.—Miss Sarah Annie Lawton has been appointed night sister. She was trained at the Stoke-on-Trent Union Hospital, later becoming staff nurse there.

Uxbridge Union Workhouse Infirmary.—Miss Winifred Lucy Wright has been appointed superintendent nurse. She was trained at Erdington Infirmary, Birmingham, and has held the post of staff nurse at Hackney Union, acting sister at Erdington Infirmary, temporary superintendent nurse at Windsor Union; she took fever training at the Isolation Hospital of the Urban District Council, Barking, and was staff nurse at the City of Birmingham Council's Infectious Hospital.

**Note.—Particulars of Appointments for insertion in this column will be welcomed.**

### Manager's Notices.

**Now Ready, "The Hospital" Index to Vol. LXI., Oct. 1916 to April 1917. Price 2½d. per copy post free**

**All letters on business matters must be addressed to The Manager, "The Hospital," 28 and 29 Southampton Street, London, W.C. 2.**

**Subscriptions,** which may commence at any time, are payable in advance. Cheques and Post-Office Orders should be crossed London County and Westminster Bank, Covent Garden Branch, and made payable to the Manager of The Hospital.

Rates of Subscription (payable in advance).

| | | |
|---|---|---|
| **UNITED KINGDOM.** | | |
| Three Months | (including Postage) | 2s. 0d |
| Six Months | do. | 4s. 0d |
| Twelve Months | do. | 6s. 6d. |
| **FOREIGN AND COLONIAL.** | | |
| Three Months | (including Postage) | 2s. 6d. |
| Six Months | do | 5s. 0d. |
| Twelve Months | do. | 8s. 8d. |

